# Computer vision in autism spectrum disorder research: a systematic review of published studies from 2009 to 2019

**DOI:** 10.1038/s41398-020-01015-w

**Published:** 2020-09-30

**Authors:** Ryan Anthony J. de Belen, Tomasz Bednarz, Arcot Sowmya, Dennis Del Favero

**Affiliations:** 1 School of Art & Design, University of New South Wales, Sydney, NSW Australia; 2 School of Computer Science and Engineering, University of New South Wales, Sydney, NSW Australia

**Keywords:** Diagnostic markers, Human behaviour, Autism spectrum disorders

## Abstract

The current state of computer vision methods applied to autism spectrum disorder (ASD) research has not been well established. Increasing evidence suggests that computer vision techniques have a strong impact on autism research. The primary objective of this systematic review is to examine how computer vision analysis has been useful in ASD diagnosis, therapy and autism research in general. A systematic review of publications indexed on PubMed, IEEE Xplore and ACM Digital Library was conducted from 2009 to 2019. Search terms included [‘autis*’ AND (‘computer vision’ OR ‘behavio* imaging’ OR ‘behavio* analysis’ OR ‘affective computing’)]. Results are reported according to PRISMA statement. A total of 94 studies are included in the analysis. Eligible papers are categorised based on the potential biological/behavioural markers quantified in each study. Then, different computer vision approaches that were employed in the included papers are described. Different publicly available datasets are also reviewed in order to rapidly familiarise researchers with datasets applicable to their field and to accelerate both new behavioural and technological work on autism research. Finally, future research directions are outlined. The findings in this review suggest that computer vision analysis is useful for the quantification of behavioural/biological markers which can further lead to a more objective analysis in autism research.

## Introduction

Visual observation and analysis of children’s natural behaviours are instrumental to the early detection of developmental disorders, including autism spectrum disorder (ASD). While a gold standard observational tool is available, there are limitations that hinder the early screening of ASD in children. Interpretative coding of child observations, parent interviews and manual testing^1^ are costly and time-consuming^2^. In addition, the reliability and validity of the results obtained from a clinician’s observations can be subjective^3^, arising from differences in professional training, resources and cultural context. Furthermore, behavioural ratings typically do not capture data from the children in their natural environments. Such limitations combined with rising incidence rates call for the development of new methods of ASD diagnosis without compromising accuracy, in order to reduce waiting periods for access to care. This is critical as diagnosis and intervention within the first few years of life can provide long-term improvements for the child and can even have greater effect on outcomes^4^.

Early behavioural risk markers of ASD have been discovered with the help of retrospective analysis of home videos^5–7^. Research studies have documented ASD-related behavioural markers that emerge within the first months of life; these include diminished social engagement and joint attention^8,9^, atypical visual attention such as difficulty during response-to-name protocol^10^, longer latencies to disengage from a stimulus if multiple ones are presented^11^, and non-smooth visual tracking^12^. Furthermore, children with ASD may exhibit atypical social behaviours such as decreased attention to social scenes, decreased frequency of gaze to faces^13^ and decreased expression of emotion. In addition, evidence suggests that differences in motor control are an early feature of ASD^14–17^.

Over the past decade, computer vision has been used in the field of automated medical diagnosis as it can provide unobtrusive objective information on a patient’s condition. A recent finding has shown that utilising computer vision methods to automatically detect symptoms can pre-diagnose over 30 conditions^18^. For example, computer vision-based facial analysis can be used to monitor vascular pulse, assess pain, detect facial paralysis, diagnose psychiatric disorders and even distinguish ASD individuals from individuals with typical development (TD) through behaviour imaging^19^. The main rationale for using computer vision for a clinical purpose would be to remove any potential bias, develop a more objective approach to analysis, increase trust towards diagnosis, as well as decrease errors related to human factors in the decision-making process. Furthermore, computer vision-based systems provide a low-cost and non-invasive approach, potentially reducing healthcare expenditures when compared to medical examinations.

Computer vision techniques have been effectively exploited in the last years to automatically and consistently assess existing ASD biomarkers, as well as discover new ones^20^. To further examine how computer vision has been useful in ASD research, a systematic review of published studies was conducted on computer vision techniques for ASD diagnosis study, therapy and autism research in general. First, eligible papers are categorised based on the quantified behavioural/biological markers. In addition, different publicly available ASD datasets suitable for computer vision research are reviewed. Finally, interesting research directions are outlined. To this end, this systematic review can serve as an effective summary resource that researchers can consult when developing computer vision-based assessment tools for automatically quantifying ASD-related markers.

## Materials and methods

### Eligibility criteria

All titles and abstracts were initially screened to include studies that meet the following inclusion criteria: (1) the study focussed on autism in humans (i.e. animal studies were excluded); (2) the study mainly focussed on the use of computer vision techniques in autism diagnosis study, therapy of autism or autism research in general; (3) the study explained how behavioural/biological markers can be automatically quantified; and (4) the study included an experiment, a pilot study or a trial with at least one group of individuals with ASD. Finally, results in the form of review, meta-analysis, keynote, narrative, editorial or magazine were excluded.

### Search process

An electronic database search of PubMed, IEEE Xplore and ACM Digital Library was conducted by including simple terms and Medical Subject Headings terms for keywords [‘autis*’ AND (‘computer vision’ OR ‘behavio* imaging’ OR ‘behavio* analysis’ OR ‘affective computing’)] in all fields (title, abstract, keywords, full text and bibliography) from January 1, 2009 to December 31, 2019. A snowballing approach was also conducted to identify additional papers. Included peer-reviewed articles followed the Preferred Reporting Items for Systematic Reviews and Meta-Analyses (PRISMA) statement^21^. Duplicates were removed and the title and abstract of each article were scanned for relevance. The full text of potentially relevant studies was assessed for eligibility considering established criteria detailed above. A PRISMA flow diagram was constructed and is shown in Appendix A.

### Data items and analysis

Identical variables in eligible studies were extracted where possible into an Excel spreadsheet: (1) quantified behavioural/biological markers; (2) application focus; (3) child diagnosis and size of participants’ groups; (4) age range of the participants or age mean and standard deviation; (5) input data and devices used; (6) computer vision method applied in the study; and (7) dataset used in the study. 94 eligible studies were categorised based on the behavioural/biological markers that were quantified.

## Results

### Overview of behavioural/biological markers used in eligible papers

The findings in this survey show that there is an increase in the number of significant contributions of computer vision methods to autism research. Over the last decade, computer vision has been used to capture and quantify different information, such as: (a) Magnetic Resonance Imaging (MRI)/functional MRI (see Table 1) (b) facial expression/emotion (see Table 2) (c) eye gaze data (see Table 3) (d) motor control/movement pattern (see Table 4) (e) stereotyped behaviours (see Table 5) and (f) multimodal data (see Table 6). Identical variables (discussed in ‘Data Items and Analysis’) were reported for each quantified information.

**Table 1:** Magnetic resonance imaging (MRI)/functional MRI (fMRI).

Reference	Focus	*N* participants	Age	Input data/device used	Method used	Dataset
Samson et al.^22^	fMRI to study the neural bases of complex non-social sound processing	15 ASD, 13 TD	ASD: 24.3 ± 6.25 TD: 23.5 ± 7.42	fMRI scans/3 T TRIO MRI system	Image processing/ICBM152 (MNI) space and 3D Gaussian Filtering	Own dataset
Abdelrahman et al.^23^	MRI for diagnosis	14 ASD, 28 TD	7-38 years	MRI scans/1.5 T Sigma MRI scanner	Mesh processing	Own dataset
Durrleman et al.^24^	MRI for biomarker detection	51 ASD, 25 TD and developmentally delayed children	18-35 months	MRI, 1.5-T GE Signa MRI scanner		^122^
Ahmadi et al.^25^	fMRI for biomarker detection	24 ASD, 27 TD		MRI scans/3T MRI scanner	Machine learning, independent component analysis	Own dataset
Chaddad et al.^26^	MRI for biomarker detection	34 ASD, 30 TD	4-24 years	MRI scans/3T MRI scanner	Texture analysis	ABIDE I dataset
Chaddad et al.^27^	MRI for biomarker detection	539 ASD, 573 TD	ASD: 17.01 ± 8.36 TD: 17.08 ± 7.72	MRI scans	Texture analysis	ABIDE I dataset
Eslami and Saeed^28^	fMRI for diagnosis	187 ASD, 183 TD		fMRI scans	Deep learning, MLP with 2 hidden layers + SVM	Four datasets (NYU, OHSU, USM, UCLA) from ABIDE-I fMRI dataset
Li et al.^29^	fMRI for diagnosis	149 ASD, 161 TD		rs-fMRI scans	Deep learning/SSAE	4 datasets (UM, UCLA, USM, LEUVEN) from ABIDE MRI dataset
Crimi et al.^30^	fMRI for diagnosis	31 ASD, 23 TD		Imaging data, GE 3T MR750 scanner	Machine Learning/Constrained Autoregressive Model	San Diego State University cohort of ABIDE II dataset
Chanel et al.^32^	fMRI for diagnosis	15 ASD, 14 TD	ASD: 28.6 ± 1.87 TD: 31.6 ± 2.61	fMRI/3T MRI scanner	Machine learning/SVM	Own dataset
Zheng et al.^34^	MRI for biomarker detection	66 ASD, 66 TD		MRI scans	multi-feature-based networks (MFN) and SVM	4 datasets (NYU, SBL, KUL, ISMMS) from ABIDE database

**Table 2:** Facial expression/emotion.

Reference	Focus	*N* participants	Age	Input data/device used	Method used	Dataset
Leo et al.^47^	Facial expression for quantitative assessment	17 ASD, 10 TD	6–13 years	Image sequences	Deep learning	Own dataset
Kalantarian et al.^36^	Facial emotion for mobile games	8 ASD	6–12 years	Mobile phone	Ensemble classification (AWS + Sighthound + Azure)	Own dataset
Kalantarian et al.^37^	Facial expression for quantitative assessment	8 ASD, 5 TD	ASD: 8.5 ± 1.85 TD: 4.4 ± 0.54 (in years)	Video, mobile phone	Histogram of Oriented Gradients (HOG) + SVM	Own dataset
Han et al.^38^	Emotional expression recognition	25 ASD		Camera	Deep learning, CNN	^128,129^
Tang et al.^39^	Automatic smile detection	11 ASD, 23 TD	6–24 months	Video, two wireless cameras	Deep learning, CNN	GENKI-4K, CelebA^132^, RCLA&NBH Smile
Daniels et al.^40^	Emotion recognition for assistive technology	23 ASD, 20 TD	6–17 years	Google Glass		n/a
Jazouli et al.^41^	Emotion recognition for assistive technology	10 ASD		3D image, Microsoft Kinect		Own dataset
Washington et al.^42^	Emotion recognition for assistive technology	14 ASD	9.57 months [3.37. 4–15]	Video/Google Glass and mobile phone	Machine learning, Histogram of Gradients (HOG) + SVM	^128,139–143^
Voss et al.^43^	Emotion recognition for assistive technology	20 ASD, 20 TD		Video/Google Glass and mobile phone	Machine learning, Histogram of Gradients (HOG) + SVM	n/a
Vahabzadeh et al.^44^	Emotion recognition for assistive technology	8 ASD	11.7–20.5 years	Video, Google Glass		n/a
Leo et al.^45^	Emotion recognition for behaviour monitoring	3 ASD		Video, Robokind R25 Robot		^128^
Pan et al.^46^	Facial emotion for behaviour analysis	2 ASD		Video, NAO robot		Own dataset
Coco et al.^48^	Facial expression analysis for diagnosis	5 ASD, 5 TD	65.38 months [15.86, 48–65 months]	Video, webcam	Deep learning, Histogram of Oriented Gradients (HOG) feature combined with a linear classifier, CNN	DISFA [24], SEMAINE [26] and BP4D [34] datasets.
Leo et al.^49^	Facial expression for quantitative assessment	17 ASD	6–13 years	Image sequences	Deep learning	Own dataset
Samad et al.^50^	3D facial imaging for physiology-based impairment detection	8 ASD, 8 TD	7–20 years	3D images, high resolution 3D facial imaging sensor, 3dMD		n/a
Leo et al.^51^	Facial expression recognition for assistive technology	1 ASD, 1 TD		Video	Deep learning, Facial Action Coding System (FACS)	Own dataset
Guha et al.^52^	Facial expression for quantitative assessment	20 ASD, 19 TD	9–14 years	Motion capture data, 6 infra-red motion-capture cameras	Deep learning, Facial Action Coding System (FACS)	Own dataset
Ahmed and Goodwin^53^	Facial expression for predicting engagement and learning performance	7 ASD	8–19 years	Video, camera	Computer Expression Recognition Toolbox	Own dataset
Harrold et al.^54^	Facial expression for assistive technology	2 ASD, 4 TD	8–10 years	Video, Apple iPad		n/a
Harrold et al.^55^	Facial expression for assistive technology	2 ASD, 4 TD	8–10 years	Video, Apple iPad		n/a
White et al.^56^	Facial emotion expression and recognition	20 ASD, 20 TD	9–12 years	3D data, Microsoft Kinect		n/a
Garcia-Garcia et al.^57^	Facial expression for learning emotional intelligence	3 ASD	8–10 years	Video, mobile phone	Affectiva SDK	n/a
Jain et al.^58^	Facial expression recognition for assistive technology	6 ASD	5–12 years	Video, webcam		^128^
Li et al.^59^	Facial attributes for ASD classification	49 ASD, 39 TD		Video, Apple iPad	Deep learning, CNN	Training: AffectNet^133^ and EmotioNet^134^ Evaluation: Own dataset
Shukla et al.^60^	Facial image analysis for diagnosis	91 ASD, 1035 NDD, 1126 TD		Image, camera	Deep learning, CNN	Own dataset

**Table 3:** Eye Gaze Data.

Reference	Focus	*N* participants	Age	Input data/device used	Method	Dataset
Pierce et al.^65^	Biomarker detection	444 subjects from 6 distinct groups		Eye tracking data, Tobii T120 eye tracker		Own dataset
Murias et al.^66^	Biomarker detection	25 ASD	24–72 months	Eye tracking data, Tobii TX300 eye tracker		Own dataset
Chawarska et al.^67^	Eye movement to determine prodromal symptoms of ASD	84 ASD	6 months	Gaze trajectories, SensoMotoric Instruments IView X RED eye-tracking system		Own dataset
Shi et al.^68^	Visual stimuli design consideration	13 ASD, 20 TD	4–6 years	Infra-red eye-tracking recording, EyeLink1000		Own dataset
Shic et al.^69^	Visual attention preference	28 ASD, 16 DD, 34 TD	20 months	Gaze patterns, SMI iView X™ RED dark-pupil 60 Hz eye-tracking system		Own dataset
Liu et al.^73^	Eye movement for diagnosis	29 ASD, 58 TD	4–11 years	Gaze data, Tobii T60 eye tracker	Machine learning, k nearest neighbours (kNN)	Own dataset
Tung et al.^61^	Eye detection	33 ASD		Video, camera		Own dataset
Balestra et al.^62^	Eye tracking to study language impairments and text comprehension and production deficits	1 ASD	25 years	Eye tracking data, Tobii 1750 eye tracker		n/a
Li et al.^63^	Identification of fixations and saccades	38 ASD, 179 TD		Eye-tracking data	Modified DBSCAN Algorithm	Own dataset
Matthews et al.^64^	Eye gaze analysis for affective state recognition	19 ASD, 19 TD	ASD: 41.05 ± 32.15 TD: 32.15 ± 9.93 (in years)	Video, Gazepoint GP3 eye-tracker	Scanpath trend analysis and arousal sensing and detection of focal attention	n/a
Campbell et al.^70^	Gaze pattern for saliency analysis	15 ASD, 13 TD	8–43 months	Gaze trajectories, SensoMotoric Instruments iView XRED eye-tracking system	Bayesian model	n/a
Syeda et al.^71^	Eye gaze for visual face scanning and emotion analysis	21 ASD, 21 TD	5–17 years	Gaze data, Tobii EyeX controller		Own dataset
Chrysouli et al.^72^	Eye gaze analysis for affective state recognition			Video, Kinect camera	Deep learning, two-stream CNN	MaTHiSis
Liu et al.^74^	Eye movement for diagnosis	Children: 20 ASD, 21 TD adults: 19 ASD, 22 intellectually disabled (ID), 28 TD	children: ASD: 7.85 ± 1.59 TD: 7.73 ± 1.51 adults: ASD: 20.84 ± 3.27 ID: 23.59 ± 3.08 TD: 20.61 ± 2.90	Eye tracking data, Tobii T60 eye tracker	Bag-of-Words (BOW) framework and SVM	^144,145^
Vu et al.^75^	Gaze pattern for diagnosis	16 ASD, 16 TD	2–10 years	Gaze data, Tobii EyeX controller	Machine learning, similarity matching + kNN	Own dataset
Jiang and Zhao^76^	Visual attention preference for diagnosis	20 ASD, 19 TD	ASD: 30.8 ± 11.1 TD: 32 ± 10.4 (in years)	Eye tracking data	Deep learning	^146^
Higuchi et al.^77^	Gaze direction for behaviour analysis	2 ASD, 2 TD		Video, camera	OpenFace Toolkit	Own dataset
Chong et al.^78^	Eye contact detection for behaviour analysis	50 ASD, 50 TD		Videos,	Deep learning	Own dataset (subset is from MMDB)
Toshniwal et al.^79^	Attention recognition for assistive technology	10 ASD, 8 NDD	12–18 years	Video, Mobile phone	Android Face Detection API	Own dataset

**Table 4:** Motor control/movement pattern.

Reference	Focus	*N* participants	Age	Input data/device used	Method used	Dataset
Dawson et al.^80^	Head movement for digital phenotyping	22 ASD, 82 TD	16–31 months	Video, iPad	Intraface, model-based object pose	Not publicly available
Martin et al.^81^	Head movement analysis	21 ASD, 21 TD	2.5–6.5 years old	Video, camera	Zface to track pitch, yaw, and roll of head movement	Not publicly available
Zunino et al.^82^	Grasping actions for diagnosis	20 ASD, 20 TD	ASD: 9.8 years TD: 9.5 years	Video, Vicon VUE video camera	Deep learning, CNN + LSTM	Publicly available
Vyas et al.^83^	Motion pattern for diagnosis			Video, Mobile Phone	R-CNN	From NODA programme of Behaviour Imaging company
Piana et al.^84^	Body movement for emotional training	10 ASD	Mean age: 9.6 years old	Video and motion capture data, Microsoft Kinect v2		n/a
Bartoli et al.^85^	Movement pattern analysis for game-based therapy	5 ASD	10–12 years old	Video, Microsoft Xbox 360 Kinect		n/a
Ringland et al.^86^	Movement pattern analysis to support therapeutic tool	15 with neurodevelopmental disorder	10–14 years old	Video, Microsoft Kinect		n/a
Magrini et al.^87^	Gesture tracking for music therapy	4 ASD	5–7 years old	Video, camera		n/a
Dickstein-Fischer and Fischer^88^	Robot-assisted therapy			Video, Penguin for Autism Behavioural Interventions (PABI)		n/a
Bekele et al.^89^	Head movement analysis for assistive technology	6 ASD, 6 TD	ASD: 4.70 ± 0.70 TD: 4.26 ± 1.05	Video, NAO Robot with 2 vertical stereo cameras	Image processing	n/a
Dimitrova et al.^90^	Movement analysis for assistive technology		7–9 years old	Video, webcam		n/a

**Table 5:** Stereotyped behaviours.

Reference	Focus	*N* participants	Age	Input data/device used	Method used	Dataset
Hashemi et al.^95^	Behaviour analysis	6 ASD, 14 TD	16–30 months	Video, iPad	IntraFace	Own dataset
Hashemi et al.^92^	Sharing interest, visual tracking, and disengagement of attention detection	3 ASD, 3 TD	6–15 months	Video, two GoPro HD cameras	Histogram of Orientated Gradients (HOG) and SVM	Own dataset
Hashemi et al.^93^	Behaviour analysis	12 ASD	5–16 months	GoPro Hero HD	HOG and SVM	Own dataset
Bidwell et al.^94^	Behaviour analysis		15–30 months	Video, Camera and Microsoft Kinect	Omron OKAO Vision Library	Multimodal Dyadic Behaviour (MMDB) Dataset
Campbell et al.^96^	Atypical orienting and attention behaviours for behavioural observation	22 ASD, 82 TD or DD	16–31 months	Tablet Device	IntraFace	Own dataset
Hashemi et al.^97^	Engagement, name-call responses, and emotional responses	15 ASD, 18 TD	16–31 months	Video, iPad	IntraFace	Own dataset
Wang et al.^98^	Attention monitoring for diagnosis	5 ASD, 12 TD		Video, two RGB cameras	Microsoft SDK	Own dataset
Bovery et al.^99^	Attention monitoring for behavioural assessment	22 ASD, 82 TD	16–31 months	Video, iPad	IntraFace	Own dataset
Rajagopalan and Goecke^100^	Self-stimulatory behaviour detection			YouTube videos	Histogram of Dominant Motions (HDM)	Self-stimulatory Behaviour Dataset, UCF101 and Weizmann Datasets
Rajagopalan et al.^101^	Self-stimulatory behaviour detection			YouTube videos	Space Time Interest Points (STIP) with Harris3D detectors in a BOW framework	Self-stimulatory Behaviour Dataset
Rajagopalan^102^	Self-stimulatory behaviour detection			YouTube videos	Motion trajectories	Self-stimulatory Behaviour Dataset, UCF101, Hollywood 2 Datasets
Winoto et al.^103^	Behaviour analysis	4 ASD, 4 TD		Microsoft Kinect v2		
Feil-Seifer and Matarić^104^	Interaction with robots for behaviour analysis	8 ASD		Video, camera	Heuristics	Own dataset
Moghadas and Moradi^105^	Interaction with robots for diagnosis	8 ASD, 8 TD	ASD: 2.1–4.1 years TD: 2.11–7.6 years	Video, RobotParrot and two cameras	Kernelised Correlation Filter (KFC) and cosine similarity and SVM	Own dataset

**Table 6:** Multimodal data.

Reference	Focus	*N* participants	Age	Input data/device used	Method used	Dataset
Egger et al.^114^	Emotion and attention analysis		16–30 months old	Video, mobile phone	IntraFace and^95^	BU-3D Facial Expression^135^ and Cohn-Kanade(CK)^127^
Rudovic et al.^110^	Autism therapy	35 ASD	3–13 years old	Synchronised video recordings of facial expressions, head and body movements, pose, and gestures, audio recordings, and autonomic physiology	Deep learning, Personalised Perception of Affect Network (PPA-net)	Own dataset - multimodal data set of children with ASC (MDCA)^147^
Chen and Zhao^106^	Attentional and image-viewing preference for diagnosis	Photo taking: 22 ASD and 23 controls image-viewing: 20 ASD and 19 controls		Photo sequence + Image and Eye fixations	Deep learning, ResNet-50 and LSTM	Own dataset (photos and eye-tracking data) and^124^
Wang et al.^107^	Mutual gaze and gesture recognition for diagnosis	2 ASD, 6 TD	Children: mean 25 months Adults: mean 25 years	Image/Two Logitech BRIO 4K Pro RGB cameras + Microsoft Kinect	Deep learning, VGG + SSD	Oxford hand and Egohands dataset
Mazzei et al.^108^	Robotic social therapy	5 ASD, 15 TD	6–12 years old			n/a
Coco et al.^109^	Face detection, landmark extraction, gaze estimation, head pose estimation and FER for behaviour analysis	8 ASD	47–93 months	Mobile tablet and Zeno R25 robot	Facial landmark Detection and Tracking: conditional local neural field^148^	Own dataset
Palestra et al.^111^	Head pose, body posture, eye contact and facial expression for robotics treatment of autism	3 ASD	8–13 years	Robokind Zeno R25 humanoid robot and a Microsoft Kinect	^149^	Own dataset
Dickstein-Fischer et al.^112^	Face recognition, head pose and eye gaze estimation for assistive technology	5 ASD	5–8 years old	Video, Penguin for Autism Behavioural Intervention (PABI)	Face Detection: Histogram of oriented gradients (HOG) + linear classifier Face recognition: LBPH Feature extraction: Regression trees Head pose estimation: Perceptive-N-Point problem	HELEN dataset
Mehmood et al.^113^	Analysis of joint attention and imitation accuracy	6 ASD, 2 TD	4–10 years old	2 NAO robots, Microsoft Kinect, and EEG		Own dataset
Peters et al.^115^	Behaviour recognition for assistive technology	2 ASD, 5 NDD	41–56 years	Two cameras, flow sensor, x-imu sensor		Own dataset
Rehg et al.^116^	Video, audio, and physiological data for behaviour analysis	121, total	15–30 months	Multimodal, cameras Microsoft Kinect, microphone, Q-sensors	Smile/Gaze Detection: Omron OKAO Vision Library + SVM	Multimodal Dyadic Behaviour (MMDB) Dataset
Liu et al.^117^	Video and audio for diagnosis	22 ASD, 21 TD	2–3 years old	Video and audio, camera		Own dataset (‘Response to Name’)
Marinoiu et al.^118^	Action and emotion for behaviour analysis	7 ASD		RGB + Depth/Microsoft Kinect v2	Deep learning	DE-ENIGMA dataset
Schwarzkopf et al.^119^	Study of larger extrastriate population receptive fields in ASD	15 ASD, 12 TD	20–48 years old	fMRI & eye gaze/3 T TIM-Trio scanner & EyeLink 1000 MRI compatible eye tracker		Own dataset

This review presents consolidated evidence on the effectiveness of using computer vision techniques in (1) determining behavioural/biological markers for diagnosis and characterisation of ASD, (2) developing assistive technologies that aid in emotion recognition and expression for ASD individuals and (3) augmenting existing clinical protocols with vision-based systems for ASD therapy and automatic behaviour analysis. The following subsections discuss in detail how each quantified marker was utilised in autism research.

#### Magnetic resonance imaging (MRI)/functional MRI (fMRI)

The need for a more quantitative approach to ASD diagnosis has pushed research towards analysing brain imaging data, such as MRI and fMRI. Generally, MRI and fMRI techniques scan different parts of the brain to provide images which are then used as input for further processing. These images have been used to determine potential biomarkers that show differences between ASD and TD subjects. For example, Samson et al.^22^ used fMRI scans to explore the differences of complex non-social sound processing between ASD and TD subjects. With increasing temporal complexity, TD subjects showed greater activity in anterolateral superior temporal gyrus while ASD subjects have greater effects in Heshl’s gyrus. Abdelrahman et al.^23^ used MRI scans to generate a 3D model of the brain and accurately calculate the volume of white matter in the segmented brain. Considering the white matter volume as a discriminatory feature in a classification step using k-nearest neighbour algorithm, their system reached an accuracy of 93%. Durrleman et al.^24^ examined MRI scans to find differences in the growth of the hippocampus in children with ASD and control subjects. Their findings suggest that group differences may be better identified by maturation speed rather than shape differences at a given age. Ahmadi et al.^25^ used independent component analysis to show that within-network connections on fMRI images of ASD subjects are lower when compared to TD subjects.

The remaining eligible studies developed new techniques for diagnosing ASD using MRI^26,27^ and fMRI^28–30^ data in the ABIDE repository. Based on their recent findings, Chaddad et al.^26^^,^^27^ demonstrated the potential of hippocampal texture features extracted from MRI scans as biomarkers for the diagnosis and characterisation of ASD. They used Laplacian-of-Gaussian filter^31^ across a range of resolution scales and performed statistical analysis to identify regions exhibiting significant textural differences between ASD and TD subjects. They identified asymmetrical difference in the right hippocampus, left choroid-plexus and corpus callosum and symmetrical difference in the cerebellar white matter.

Some of the techniques are based on conventional machine learning techniques, such as Support Vector Machines (SVM). For example, Chanel et al.^32^ used a multivariate pattern analysis approach in two different fMRI experiments with social stimuli. The method, based on a modified version of SVM Recursive Feature Elimination algorithm^33^, is trained independently and then combined to obtain a final classification output (e.g. ASD or TD). Their results revealed classification accuracy of between 69% and 92.3%. Crimi et al.^30^ used a constrained autoregressive model followed by an SVM to differentiate individuals with ASD from TD individuals. Zheng et al.^34^ constructed multi-feature-based networks (MFN) and SVM to classify individuals of the two groups. Their results showed that using MFN significantly improved the classification accuracy by almost 14% compared to using morphological features. Their findings also demonstrated that variations in cortico-cortical similarities can be used as biomarkers in the diagnostic process.

Deep learning techniques have also been proposed for automating ASD diagnosis by extracting discriminative features from fMRI data and feeding them to a classifier^28^. In order to increase the number of training samples and avoid overfitting, Eslami and Saeed^28^ used Synthetic Minority Over-Sample (SMOTE)^35^. They also investigated the effectiveness of the features extracted using an SVM classifier. Their model achieved more than 70% classification accuracy for four fMRI datasets, with highest accuracy of 80%. Attaining similar performance, Li et al.^29^ adopted a deep transfer learning neural network model for ASD diagnosis. Compared to traditional models, their approach led to improved performance in terms of accuracy, sensitivity, specificity and area under receiver operating characteristic curve.

#### Facial expression/emotion

Emotion classification focusses on the development of algorithms that produce an emotion label (e.g. happy or sad) from a face in a photo or a video frame. Recent advances in the field of computer vision have contributed to the development of various emotion classifiers that can potentially play a significant role in mobile screening and therapy for ASD children. However, most classifiers are biased towards neurotypical adults and can fail to generalise to children with ASD. To address this, Kalantarian et al.^36,37^ presented a framework for semi-automatic label frame extraction to crowdsource labelled emotion data from children. The labels consist of six emotions: disgust, neutral, surprise, scared, angry and happy. To improve the generalisation of expression recognition models to children with ASD, Han et al.^38^ presented a transfer learning approach based on a sparse coding algorithm. Their results showed that their method can more accurately identify the emotional expression of children with ASD. Tang et al.^39^ proposed a convolutional neural networks-based (CNN) method for smile detection of infants in mother–infant interaction. Their results showed that their approach can achieve a mean accuracy of 87.16% and F1-score of 62.54%.

Several papers have focussed on using computer vision to develop assistive technologies for ASD children^40–43^. For example, researchers^40,42,43^ developed and evaluated a wearable assistive technology to help ASD children with emotion recognition. Vahabzadeh et al.^44^. provided initial evidence for the potential of wearable assistive technologies to reduce hyperactivity, inattention and impulsivity in school-aged children, adolescents and young adults with ASD. Leo et al.^45^ and Pan et al.^46^ developed an automatic emotion recognition system in robot-children interaction for ASD treatment. Their results suggest that computer vision could help to improve the efficiency of behaviour analysis during interactions with robots.

Most research mainly focusses on qualitative recognition of facial expressions. This is due to the fact that computational approach on facial expression analysis is an emerging research topic. There are a few attempts to automatically quantify facial expression production of ASD children^47–52^. For example, Leo et al.^47^ proposed a framework to computationally analyse how ASD and TD children produce facial expression. Guha et al.^52^ investigated differences in the overall and local facial dynamics of TD and ASD children. Their observations showed that there is reduced complexity in the dynamic facial behaviour of ASD children arising primarily from the eye region. Computer vision has also been used to predict engagement and learning performance. For example, Ahmed and Goodwin^53^ analysed facial expressions from video recordings obtained when kids interacted with a computer-assisted instruction programme. Their results showed that emotional and behavioural engagement can be quantified automatically using computer vision analysis.

Harrold et al.^54^^,^^55^ developed a mobile application that allows children to learn emotions with instant feedback on performance through computer vision. White et al.^56^ presented results which showed that children with ASD found their system to be acceptable and enjoyable. Similar to this approach, Garcia-Garcia et al.^57^ presented a system that incorporates emotion recognition and tangible user interfaces to teach children with ASD to identify and express emotions. Jain et al.^58^ proposed an interactive game that can be used for autism therapy. The system tracks facial features to recognise the facial expressions of the participant and to animate an avatar. Developed as a game, the system attempts to teach kids how to recognise and express emotions through facial expressions.

A deep learning approach has also been applied to recognise developmental disorders through facial images. For example, Li et al.^59^ introduced an end-to-end CNN-based system for ASD classification using facial attributes. Their results show that different facial attributes are statistically significant and improve classification performance by about 7%. A deep convolutional neural network (DCNN) for feature extraction followed by an SVM for classification has been trained by Shukla et al.^60^ to detect whether a person in an image has ASD, cerebral palsy, Down syndrome, foetal alcohol spectrum syndrome, progeria or other intellectual disabilities. Their results indicate that their model has an accuracy of 98.80% and performs better than average human intelligence in distinguishing between different disorders.

#### Eye gaze data

Analysing attention and psychological factors encoded in eye movements of individuals could help in ASD diagnosis. Computer vision has been used to automatically analyse children’s gaze and distinguish ASD-related characteristics present in a video^61^. Research has shown that there is a significant difference in gaze patterns between children with ASD and TD. Eye tracking technology provides automatic assessment of gaze behaviour in different contexts. For example, Balestra et al.^62^ showed that it can be used to study language impairments, text comprehension and production deficits. In addition, it can be used to identify fixation and saccades^63^, recognise affective states^64^ and even reveal early biomarkers associated with ASD^65,66^. Furthermore, eye tracking can be used to detect saliency differences between ASD and TD children. Researchers^67–70^ showed that there is a difference in preference for both social and non-social images. This finding is consistent with a similar published study of Syeda et al.^71^, which examined face scanning patterns in a controlled experiment. By extracting and analysing gaze data, the study revealed that children with autism spend less time looking at core features of faces (e.g. eyes, nose and mouth). Chrysouli et al.^72^ proposed a deep learning-based technique to recognise the affective state (e.g. engaged, bored, frustrated) of an individual (e.g. ASD, TD, etc.) from a video sequence.

Building upon the knowledge of previous research, several studies have concentrated on using visual attention preference of children with ASD for diagnosis. For example, Liu et al.^73,74^ proposed a machine learning-based system to capture discriminative eye movement patterns related to ASD. They also presented a comprehensive set of effective feature extraction methods, prediction frameworks and corresponding scoring frameworks. Vu et al.^75^ examined the impact of visual stimuli and exposure time on the quantitative accuracy of ASD diagnosis. They showed that using a ‘social scene’ stimulus with 5-s exposure time has the best performance at 98.24%. By also using visual attention preference, Jiang and Zhao^76^ leveraged recent advances in deep learning for superior performance in ASD diagnosis. In particular, they used a DCNN and SVM to achieve an accuracy of 92%. Higuchi et al.^77^ developed a novel system that provides visualisation of automatic gaze estimation and allows for experts to perform further analysis.

Most of the studies have been conducted in a highly controlled environment in which the subjects were asked to view a screen for a short period of time. Recently, Chong et al.^78^ presented a novel deep learning architecture for eye contact detection in natural social interactions. In their study, eye contact detection was performed during adult–child sessions in which the adult wears a point-of-view camera. Their results showed significant improvement over existing methods, with a reported precision and recall of 76% and 80%, respectively. Toshniwal et al.^79^ proposed an assistive technology that tracks attention using mobile camera and uses haptic feedback to recapture attention. Their evaluation study with users with various intellectual disabilities showed that it can provide better learning with less intervention.

#### Motor control/movement pattern

The use of computer vision has also shown potential for a more precise, objective and quantitative assessment of early motor control variations. For example, Dawson et al.^80^ used computer vision analysis to analyse differences in midline head postural control, as reflected in the rate of spontaneous head movements between toddlers with ASD versus those without ASD. Their study followed a response-to-name protocol where a series of social and non-social stimuli (i.e. in the form of a movie) were shown on a smart tablet while the child sat on a caregiver’s lap. During the protocol, the examiner, standing behind the child, calls the child’s name and the child’s reaction is recorded using the smart tablet. Afterwards, a fully automated computer vision algorithm detects and tracks 49 facial landmarks and estimates head pose angles. Their study revealed that toddlers with ASD exhibited a significantly higher rate of head movement compared to their typically developing counterparts. Using the same approach, Martin et al.^81^ examined head movement dynamics of a cohort of children. They found that there is an evident difference in lateral (yaw and roll) head movement between children with ASD and TD children.

Deep learning has also been employed to develop novel screening tools that analyse gestures captured in video sequences. For example, Zunino et al.^82^ used CNN to extract features, followed by a long short-term memory (LSTM) model with an attentional mechanism. They demonstrated that it is possible to determine whether a video sequence contains grasping action performed by ASD or TD subjects. In another study, Vyas et al.^83^ estimated children’s pose over time by retraining a state-of-the-art pose estimator (2D Mask R-CNN) and trained a CNN to categorise whether a given video clip contains a typical (normal) or atypical (ASD) behaviour. Their approach with an accuracy of 72% outperformed conventional video classification approaches.

Computer vision has also been used to develop motion-based touchless games for ASD therapy. For example, Piana et al.^84^ conducted an evaluation study of a system designed for helping ASD children to recognise and express emotions by means of their full-body movement captured by RGB-D sensors. Their results showed that there is an increase in task (recognition) accuracy from the beginning to the end of training sessions. Bartoli et al.^85^ showed the effectiveness of using embodied touchless interaction to promote attention skills during therapy sessions. Similarly, Ringland et al.^86^ developed SensoryPaint that allows whole-body interactions and showed that it is a promising therapeutic tool. Magrini et al.^87^ developed an interactive vision-based system which reacts to movements of the human body to produce sounds. Their system has been evaluated by a team of clinical psychologists and parents of young patients.

Computer vision has also been used to develop robot-mediated assistive technologies for ASD therapy. Dickstein-Fischer and Fischer^88^ developed a robot, named PABI (Penguin for Autism Behavioural Interventions), with augmented vision to interact meaningfully with an autistic child during therapy. Similarly, Bekele et al.^89^ developed a robot with augmented vision to automatically adapt itself in an individualised manner and to administer joint attention prompts. Their study suggests that robotic systems with augmented vision may be capable of enhancing skills related to attention coordination. This confirms an earlier study of Dimitrova et al.^90^ where adaptive robots showed potential for educating children in various complex cognitive and social skills that eventually produce a substantial development impact.

#### Stereotyped behaviours

In the context of autism research, atypical behaviours are assessed during screening using different clinical tools and protocols. For example, Autism Observation Scale for Infants (AOSI) consists of a set of protocols that is designed to assess specific behaviours^91^. During the last decade, research has been growing towards behavioural imaging to create new capabilities for the quantitative understanding of behavioural signs, such as those outlined in AOSI. For example, Hashemi et al.^92,93^ examined the potential benefits that computer vision can provide for measuring and identifying ASD behavioural signs based on two components of AOSI. In particular, they developed a computer vision tool to assess: (1) disengagement of attention: the ability of kids to disengage their attention from one of two competing visual stimuli and (2) visual tracking, to visually follow a moving object laterally across the midline. Similarly, computer vision analysis has also been explored to automatically detect and analyse atypical attention behaviours in toddlers in a response-to-name protocol. A proof of concept system that used marker-less head tracking was presented by Bidwell et al.^94^ and scalable applications were developed by Hashemi et al.^95^, Campbell et al.^96^ and Hashemi et al.^97^. The latter systems run on a mobile application designed to elicit ASD-related behaviours (e.g. social referencing, smiling while watching a movie and pointing) and use computer vision analysis to automatically code behaviours related to early risk markers of ASD. When compared to a human analyst, computer vision analysis was found to be as reliable in predicting child response latency. Using the response-to-name protocol, Wang et al.^98^ proposed a non-contact vision system that achieved an average classification score of 92.7% for assistant screening of ASD. The results of the mentioned studies show that computer vision tools can capture critical behavioural observations and potentially augment clinical behavioural observations when using AOSI. Bovery et al.^99^ also used a mobile application and movie stimuli to measure attention of toddlers. They used computer vision algorithms to detect head and iris positions and determine the direction of attention. Their results showed that toddlers with ASD paid less attention to the movie, showed less attention to the social as compared to the non-social visual stimuli and often directed their attention to one side of the screen.

Behaviours other than those outlined by AOSI have also been quantified using computer vision. For example, self-stimulatory behaviours refer to stereotyped, repetitive movements of body parts. Also known as ‘stimming behaviours’, these behaviours are often manifested when a person with autism engages in actions like rocking, pacing or hand flapping. Researchers^100–102^ have introduced a dataset with stimming behaviours and used computer vision to determine if these behaviours exist in a video stream. Another quantified behaviour is social interaction and communication among individuals with ASD. Winoto et al.^103^ developed an unobtrusive sensing system to observe, sense and annotate behavioural cues which can be reviewed by specialists and parents for better tailored assessment and interventions. Similarly, children’s responses when interacting with robots have been quantified using computer vision techniques. Feil-Seifer and Matarić^104^ showed that computer vision can be used to study behaviours of ASD children towards robots during free-play settings. Moghadas and Moradi^105^ proposed a computer vision approach to analyse human-robot interaction sessions and to extract features that can be used for ASD diagnosis.

#### Multimodal data

Over the last decade, there has been increasing interest in incorporating multiple behavioural modalities to achieve superior performance and even outperform previous state-of-the-art methods that utilise only a single modality for ASD screening. For example, Chen and Zhao^106^ proposed a privileged modality framework that integrates information from two different tasks; (1) photo taking task where subjects freely move around the environment and take photos and (2) image-viewing task where their eye movements are recorded by an eye-tracking device. They used CNN and LSTM to integrate features extracted from these two tasks for more accurate ASD screening. Their results showed that the proposed models can achieve new state-of-the-art results. They also demonstrated that utilising knowledge across the two modalities dramatically improved performance by more than 30%.

Wang et al.^107^ presented a standardised screening protocol, namely Expressing Needs with Index Finger Pointing (ENIFP), to assist in ASD diagnosis. The protocol is administered in a novel non-invasive system trained using deep learning to automatically capture eye gaze and gestures of the participant. Their results showed that the system can record the child’s performance and reliably check mutual attention and gestures during the ENIFP protocol. Computer vision techniques have also been used during robotic social therapy sessions proposed by Mazzei et al.^108^.

Computer vision systems that incorporate multimodal information have also been used to detect behavioural features during interaction with a humanoid robot. For example, Coco et al.^109^. proposed a technological framework to automatically build a quantitative report that could help therapists to better achieve either ASD diagnosis or assessment tasks. Furthermore, computer vision has been used to address autism therapy through social robots that automatically adapt their behaviours. For example, researchers^110–113^ have presented systems that simultaneously include eye contact, joint attention, imitation and emotion recognition for an intervention protocol for ASD children. Egger et al.^114^ presented the first study showing the feasibility of computer vision techniques to automatically code behaviours in natural environments. Another assistive technology was introduced by Peters et al.^115^. to assist people with cognitive disabilities in brushing teeth. It uses behaviour recognition and a machine learning network to provide automatic assistance in task execution.

Rehg et al.^116^. proposed a new action recognition dataset for analysis of children’s social and communicative behaviours based on video and audio data. Their preliminary experimental results demonstrated the potential of this dataset to drive multi-modal activity recognition. Similarly, Liu et al.^117^ proposed a ‘Response-to-Name’ dataset and a multimodal ASD auxiliary screening system based on machine learning. Marinoiu et al.^118^ introduced one of the largest existing multimodal datasets of its kind (i.e. autistic interaction rather than genetic or medical data). They also proposed a fine-grained action classification and emotion prediction task recorded during robot-assisted therapy sessions of children with ASD. Their results showed that machine-predicted scores align closely with human professional diagnosis.

Computer vision has also been applied to multimodal data, such as fMRI and eye gaze information, in order to test differences in response selectivity of the human visual cortex between individuals with ASD and TD. Schwarzkopf et al.^119^. have shown that sharper spatial selectivity in visual cortex is not characterised in ASD individuals.

### Datasets used in eligible papers

The dataset requirement typically depends on the target behavioural/biological marker and the computer vision methods to be employed. In this section, the publicly available datasets used by eligible papers are reviewed and those with autistic samples are focussed on.

#### Magnetic resonance imaging datasets

Autism Brain Imaging Data Exchange (ABIDE) initiative has aggregated functional and structural brain imaging data collected from different laboratories to accelerate understanding of the neural basis of autism. ABIDE I represents the first ABIDE initiative^120^. This effort yielded a total of 1112 records (sets of magnetic resonance imaging (MRI) and functional MRI), including 539 from individuals with ASD and 573 from TD individuals. ABIDE II was established to further promote discovery of brain connectome in ASD^121^. It consists of 1114 records from 521 individuals with ASD and 593 TD individuals. Hazlett et al.^122^ conducted an MRI study with 51 children with ASD and 25 control children (including both developmentally delayed and TD children) between 18 and 35 months of age.

#### Autism spectrum disorder detection dataset

This dataset consists of a set of video clips of reach-to-grasp actions performed by children with ASD and TD^82^. In the protocol, children were asked to grasp a bottle and perform different subsequent actions (e.g. placing, pouring, passing to pour, and passing to place). A total of 20 children with ASD and 20 TD children participated in the study.

#### DE-ENIGMA dataset

DE-ENIGMA dataset is a free, large-scale, publicly available multi-modal (e.g. audio, video, and depth) database of autistic children’s interactions that is suitable for behavioural research^123^. A total of 128 children on the autism spectrum participated in the study. During the experiment, children within each age group were randomly assigned to either a robot-led or a researcher/therapist-led teaching intervention which was implemented across multiple short sessions. This dataset includes ~13 TB of multi-modal data, representing 152 h of interaction. Furthermore, 50 children’s data have been annotated by experts for emotional valence, arousal, audio features and body gestures. The annotated data are in effect ready for future autism-focussed machine learning research.

#### Multimodal behaviour dataset

The Multimodal Dyadic Behaviour (MMDB) dataset is a unique collection of 160 multimodal (video, audio and physiological) recordings and annotations of the social and communicative behaviours of 121 children aged 15–30 months, gathered in a protocol known as the Rapid-ABC sessions^116^. This play protocol is an interactive assessment (3–5 min) consisting of five semi-structured play interactions in which the examiner elicits social attention, interaction and non-verbal communication from the child.

#### Saliency4ASD dataset

Saliency4ASD Grand Challenge aims to align the visual attention modelling community around the application of ASD diagnosis and to provide an open dataset of eye movements recorded from children with ASD and TD. The database consists of 300 images with various animals, buildings, natural scenes and combinations of different visual stimuli^124^. Each image has corresponding eye-tracking data collected from 28 participants.

#### Self-stimulatory behaviour dataset

Due to the lack of a database containing self-stimulatory behaviours, Rajagopalan et al.^101^ searched for and collected videos on public domain websites and video portals (e.g. YouTube). They classified the videos into three categories: arm flapping, head banging and spinning. Compared to other datasets, their dataset is recorded in natural settings. The dataset contains 75 videos with an equal number of videos for each category.

#### Other datasets

Until recently, autism datasets have been relatively small when compared to other datasets in which machine learning has seen tremendous application. As a result, earlier published research has resorted to using a subset of videos of neurotypical individuals from human action recognition datasets [UCF101^125^, Weizmann^126^], facial expression datasets [Cohn-Kanade(CK)^127^, CK+^128^, FERET^129^, Hollywood2^130^, HELEN^131^, CelebA^132^, AffectNet^133^, EmotioNet^134^, BU-3D Facial Expression^135^] and gesture recognition datasets [Oxford Hand Dataset^136^, Egohands^137^] to help train systems that analyse autistic behaviours.

### Limitations

This review has some limitations: one is linked to the number of included papers and the other to the quality of papers included. Although it has been attempted to make the review as inclusive as possible through the PRISMA checklist, there are studies that might not have been included because of the chosen keywords and time period used. Nevertheless, as far as is known, this is the first systematic review of the current state of computer vision approaches in autism research.

Being a relatively new field of research, some published papers have few longitudinal studies or included small cohorts of participants, thus the quality of the results may change as more clinical trials are conducted. Nonetheless, this systematic review suggests that these advances in computer vision are applicable in the ASD domain and can stimulate further research in using computer vision techniques to augment existing clinical methods. However, these approaches require further evaluation before they can be applied in clinical settings.

## Discussion

In this work, a systematic review has been provided on the use of computer vision techniques in autism research in general. Although there have been considerable studies on this area, different factors such as controlled experiments in a clinical setting mean that quantification of human behaviours in real scenarios remains challenging in the context of understanding image or video streams. In this paper, publicly available datasets relevant to behaviour analysis have also been reviewed, in order to rapidly familiarise researchers with datasets applicable to their field and to accelerate both new behavioural and technological work on autism. The primary conclusion of this study on computer vision approaches in autism research are provided below:

1. Different behavioural/biological markers have already been quantified, to some extent, using computer vision analysis with comparable performance to a human analyst.
2. For feature extraction and classification tasks, deep learning-based approaches have shown superior performance when compared to traditional computer vision approaches.
3. The growing number of large-scale publicly available datasets provides the required scale of data needed for furthering machine learning and deep learning developments.
4. Multimodal methods attain superior performance by combining knowledge across different modalities.

In the current state of the art, it is evident that computer vision analysis is useful for the quantification of behavioural/biological markers that can further lead to a non-invasive, objective and automatic tool for autism research. It can also be used to provide effective interventions using robots with augmented vision during therapies. In addition, it can be used to develop technologies that assist individuals with ASD in certain tasks, such as emotion recognition.

To date, most published studies are related to the use of computer vision in a clinical setting. However, in complex scenes outside of clinical protocols, there are many issues with feature learning in single or even multimodal data. In addition, it is challenging to compare the performance of the eligible studies due to the lack of benchmarked datasets that researchers have ‘agreed’ on for the use of deep learning^138^. Until recently, there have been no large-scale datasets that researchers could use to compare their results. Given the current state of research, researchers in this area should address the following problems:

1. Multimodal approaches based on multimodal fusion methods. In current research, most studies have focussed on RGB data from image or video streams. However, an increasing number of studies has shown that superior performance can be achieved through a combination of multimodal information.
2. Researchers should agree to work on a benchmark dataset and evaluate their models on them for more reliable comparison of performance. The datasets reviewed in this paper serve as a starting point for researchers to use in computer vision research. Experts can borrow knowledge gained from existing state-of-the-art human activity recognition models trained on neurotypical individuals, apply them to these datasets, and build models that can generalise to individuals with ASD.
3. Computer vision approaches that address fully unconstrained scenarios. Most published studies require participants to be in clinical settings that typically do not capture data from the children in their natural environments.
4. Longitudinal studies or a collection of a large cohort of individuals with ASD and TD individuals should be conducted to evaluate the performance of succeeding computer vision systems. This requires a careful and systematic empirical validation to ensure their accuracy, reliability, interpretability and true clinical utility. This would help determine if these systems can generalise across different participant groups (e.g. multiple ages, cultural differences) and demonstrate fairness and unbiasedness.
5. It is also important to gain a deeper understanding of human factors, user experience and ethical considerations surrounding the application of vision-based systems. This would help develop usable and useful systems and determine if these systems can really be used to augment existing behavioural observations in a clinical setting.

## Supplementary information

Appendix A: PRISMA 2009 flow diagram: computer vision in autism spectrum disorder research: a systematic review of published studies from 2009 to 2019
