# Supplementary material for: Computer vision in autism spectrum disorder research: a systematic review of published studies from 2009 to 2019
Source: Transl Psychiatry. 2020 Sep 30;10:333. doi: 10.1038/s41398-020-01015-w (PMC7528087; doi:10.1038/s41398-020-01015-w)
Supplement: Supplementary file 1 — Appendix A: PRISMA 2009 flow diagram: computer vision in autism spectrum disorder research: a systematic review of published studies from 2009 to 2019 [file 41398_2020_1015_MOESM1_ESM.docx]

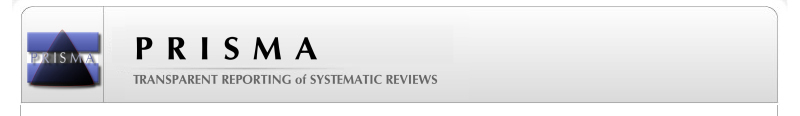
**Appendix A: PRISMA 2009 Flow Diagram:**

**Computer Vision in Autism Spectrum Disorder Research: A Systematic Review of Published Studies from 2009-2019**

Records identified through database searching through PubMed, IEEEXplore, and ACM Digital Library from 1/1/2009 to 31/12/2019
(n = 919)

## Identification

Records excluded
(n = 656)

Records screened
(n = 740)

Records after duplicates removed
(n = 740)

Full-text articles assessed for eligibility
(n = 84)

## Screening

## Eligibility

## Included

Studies included in qualitative synthesis
(n = 94)

Full-text articles included through snowballing approach^47,65-69,73,95,110,114^
(n = 10)
